# Effects of hydroxyurea on fertility in male and female sickle cell disease patients. A systemic review and meta-analysis

**DOI:** 10.1371/journal.pone.0304241

**Published:** 2024-06-07

**Authors:** Sarah Sewaralthahab, Lujain A. Alsubki, Maram S. Alhrabi, Abdulrahman Alsultan

**Affiliations:** 1 Department of Internal Medicine, College of Medicine, King Saud University, Riyadh, Saudi Arabia; 2 Oncology Center, King Saud University Medical City, Riyadh, Saudi Arabia; 3 Division of Reproductive Endocrinology and Infertility, Department of Obstetrics and Gynecology, King Faisal Specialist Hospital and Research Centre, Riyadh, Saudi Arabia; 4 Department of Pediatrics, College of Medicine, King Saud University, Riyadh, Saudi Arabia; University of Illinois at Chicago, UNITED STATES

## Abstract

**Background:**

Evidence supports the benefits of hydroxyurea (HU) in adults with sickle cell disease (SCD), but reservations remain due to long-term concerns of fertility. Retrospective analysis of clinical records of SCD patients (haemoglobin SS genotype) have identified gender-related differences in disease progression. This could inform risk stratification during SCD at diagnosis with the possibility to guide therapeutic decisions.

**Methods:**

This systemic review and meta-analysis evaluated fertility parameters in both children (aged ≥ 6 years) and adults with SCD receiving HU therapy. Studies were sourced from PubMed and EMBASE from inception to July 2023. A total of 160 potentially relevant articles were identified.

**Results:**

Four studies were included that evaluated the effects of HU on sperm parameters in males. A further 4 studies assessed anti-mullerian hormone (AMH) levels and ovarian reserves in females. Differences from baseline values were used to identify compromised fertility. Amongst males, HU treatment negatively impacted the concentration of spermatozoa (MD = -15.48 million/mL; 95% CI: [-20.69, -10.26]; p< 0.001), which continued following treatment cessation (MD = -20.09 million/mL; 95% CI: [-38.78, -1.40]; P = 0.04). HU treatment also led to lower total sperm counts (MD = -105.87 million; 95% CI: [-140.61, -71.13]; P< 0.001) which persisted after treatment (MD = -53.05 million; 95% CI: [-104.96, -1.14]; P = 0.05). Sperm volume, initial forward motility and morphology were unaffected by HU treatment. In females, HU treatment decreased the mean AMH levels 1.83 (95% CI [1.42, 2.56]. A total of 18.2.% patients treated with HU showed reduced ovarian reserves.

**Interpretation & conclusions:**

This systemic review and meta-analysis suggest that the use of HU for SCD impacts seminal fluid parameters in males and can diminish AMH levels and ovarian reserves in females.

## Introduction

Sickle cell disease (SCD) remains one of the most common inherited disorders globally [1]. In SCD individuals, abnormal sickle haemoglobin (HbS) forms polymers within red blood cells upon de-oxygenation, impeding blood flow leading to inflammation, vasculopathy and chronic hemolysis [1–4]. SCD predominately affects individuals originating from sub-Saharan Africa, the Mediterranean, Arab countries, India, the Caribbean and South America, as well as African‐Americans [5–8]. Based on current statistics, the estimated global birth rate is 515,000 individuals per-year. This translates to approximately 382 cases per 100,000 live births [8].

Hydroxyurea (HU) remains a widely available and clinically effective therapy for SCD [9–12]. HU was initially reserved for adult patients with clinical complications, but is now recommended to all SCD patients from 9 months of age, regardless of disease severity [11, 13–23]. A substantial body of evidence documents the benefits of HU with acceptable short- and long-term toxicity profiles, but concerns regarding its long-term safety persist, particularly regarding fertility [24]. As a ribonucleotide reductase inhibitor, HU can impair DNA synthesis, damaging actively dividing cells including gametes. In males, HU has been suggested to impact sperm counts for over 1 year following the cessation of therapy [24–26]. In females, diminished ovarian reserves and a higher risk of pregnancy associated teratogenicity has been documented [27]. The use of HU during pregnancy has also been reported to increase the risk of miscarriage, stillbirths and low birth weights [27]. Exposing SCD children to HU from an early age may therefore compromise their fertility and reproductive capability [17, 24–35].

Information on the effects of HU on human spermatogenesis and female reproductive capacity remain limited. This systemic review and meta-analysis combined data from publications in this area to determine the current understanding of fertility risks. We further describe recommendations and interventions based on the outcome of these analyses.

## Materials and methods

### Search strategy, sources and selection process

A systematic literature review was performed according to the guidelines of the Preferred Reporting Items for Systematic Reviews and Meta-Analyses (PRISMA) [36]. Articles published from inception to July 2023 were searched in PubMed and EMBASE. Terms used in the research for primary endpoints were ““sickle cell disease” and “infertility” and “hydroxyurea”; “sickle cell disease” and “fertility” and “hydroxyurea”; “sickle cell anaemia” and “hydroxyurea” and “infertility”; “sickle cell disease” and “hydroxycarbamide” and “infertility”; “sickle cell disease” and “hydroxycarbamide/hydorxyurea” and “ovarian reserve” or combination of the terms “sickle cell anaemia” and “hydroxycarbamide” and “infertility”. Our research focused on primary fertility outcomes stratified by male or female gender.

### Study characteristics

Inclusion criteria were as follows: (i) Studies published in English from inception to the present day; (ii) Studies subjects (aged ≥ 6 years), prospective and retrospective cohort studies reporting frequency of outcomes of interests (semen parameters and female infertility events) stratified by HU therapy. Outcomes were measured according to frequency of events. All HU therapies were considered as one type of therapy. HU is conventionally administered by the oral route.

Case reports, reviews, animal studies, duplications and studies on very young patients (aged < 6 years) were excluded. Given that abnormal semen parameters and AMH values are common in SCD patients, these were not deemed as exclusion criteria for study.

### Effect measures

Male studies were assessed for the effects of HU on the volume of ejaculate (mL), spermatozoa concentration (millions/mL), total sperm count (millions), initial forward motility (% of motile), spermatozoa morphology (% of normal), and vitality (% of living sperm). Female studies were assessed for the effects of HU on Anti-Müllerian hormone (AMH; ng/mL), normal ovarian reserve (follicles per-ovary) and diminished ovarian reserve. In the pubertal and post-pubertal age groups, high AMH blood levels are deemed as over 4.0 ng/ml, normal levels: 1.5–4.0 ng/ml, low-normal levels: 1.0–1.5 ng/ml, and low levels: 0.5–1.0 ng/ml. Similarly, normal ovarian reserve in these groups is between 4 and 8 follicles per ovary; diminished ovarian reserve is deemed as ≤ 4 follicles per ovary.

### Data extraction

Two reviewers extracted the data independently. Key characteristics including author name, year of publication, study design, type of study, number of HU treated patients, sperm volume (mL), spermatozoa concentration (millions/mL), initial forward motility (% of motile), spermatozoa morphology (% of normal), anti-Müllerian levels (ng/mL), ovarian reserve (follicles per-ovary) and serum levels of Follicle-stimulating hormone (FSH) were collected. Data were reported as the mean difference ± SD where applicable. Discrepancies in analyses were resolved by a third reviewer.

### Assessment of study quality

Quality and bias assessments of eligible studies were performed independently by two reviewers. The Newcastle-Ottawa Scale (NOS) was used to assess the quality and risk of bias. The scale is designed to assess [1] selection; [2] comparability and [3] outcomes, divided across nine specific items. The maximum score on NOS was 9. Scores ≥ 7 were deemed high quality with a low risk of bias. Scores <5 were categorised as low quality with a high risk of inherent bias. Scores between these values were rated as moderate quality. Study quality was independently conducted by two investigators. Discrepancies were solved by discussion with a third investigator. Average NOS scores were tabulated and are shown in Table 3.

### Statistical analysis

Continuous data are presented as the mean difference. Dichotomous data are presented as proportions (OR), with corresponding 95% confidence intervals (CI). P-values ≤ 0.05 were considered statistically significant. Statistical heterogeneity amongst studies was evaluated using the Chi-square test (Cochrane Q test) followed by the chi-square statistic. Cochrane Q, was used to calculate I-squared values according to the equation: I^2^ = ((Q-df)/Q) x100%. A chi-square p value ≤0.1 was considered as significant heterogeneity. I^2^ values ≥ 50% were indicative of high heterogeneity. The fixed effect model for the meta-analysis was used if no heterogeneity was present. The random effect DerSimonian-Liard meta-analysis model was used when significant heterogeneity was observed. RevMan (RevMan 5.4.1) and Jamovi software were used to perform the meta-analyses as previously described [37, 38].

## Results

### Search output

**Fig 1:**
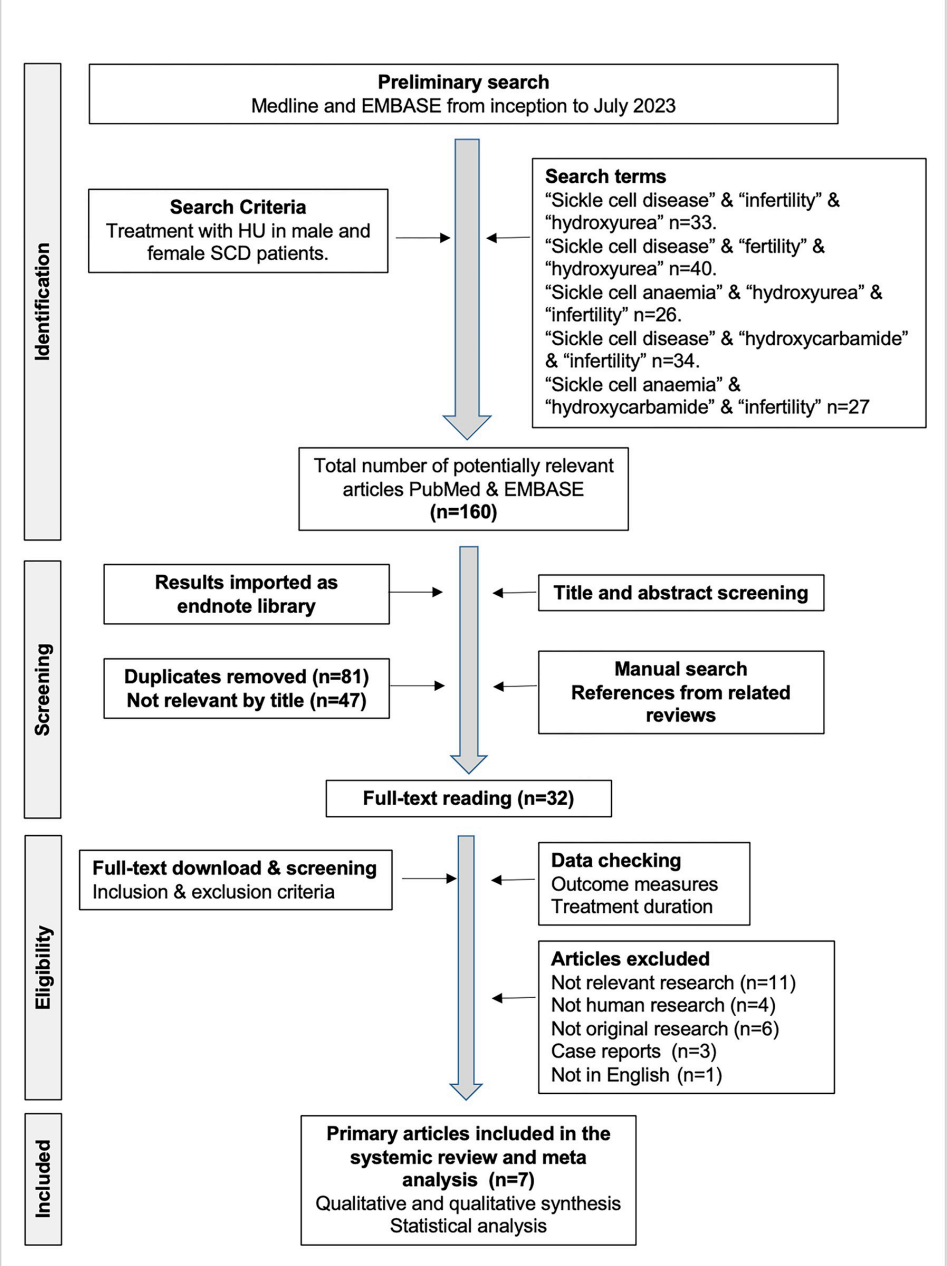
Flow diagram depicting the search strategy.

Combination of the search terms “sickle cell disease” and “infertility” and “hydroxyurea” yielded 33 papers for assessment. Combination of the search terms “sickle cell disease” and “fertility” and “hydroxyurea” yielded 40 papers. Combination of the terms “sickle cell anaemia” and “hydroxyurea” and “infertility” yielded 26 papers. Combination of terms “sickle cell disease” and “hydroxycarbamide” and “infertility” yielded 34 papers. Combination of the terms “sickle cell anaemia” and “hydroxycarbamide” and “infertility” yielded 27 papers. Combination of the terms “sickle cell anaemia” and “hydroxycarbamide” and “ovarian reserve” yielded 7 papers for review. A total of 167 articles were initially retrieved from PubMed and EMBASE. After applying the exclusion criteria, the full texts of 34 potentially relevant studies were reviewed. A total of 26 were excluded due to lack of relevant research (n = 12), non-human research (n = 4), not original research (n = 6), case reports (n = 3) and articles not in English (n = 1). In total, 8 were finally included for meta-analysis (Fig 1; Tables 1 and 2).

**Table 1:** Summary of studies in which male sickle cell anaemia patients were treated with hydroxyurea.

Study ID		Berthaut 2008		Berthaut 2017	Joseph 2021		Sahoo 2017
Intervention	(Time of assessment)	During treatment (2 to 10 years)	After treatment (stoppage)	During treatment (after 6 months of initiation)	HU-naıve patients	HU-exposed (after HU withdrawal)	HU therapy group (during)
** Mean Age**	**(y)**	33.62		25.8	17.0		33.2
**Volume of ejaculate (mL)**	**Mean change**	-0.4	-0.09	0.16	2.54	4	
	**SD change**	1.00356	1.81554	1.18493	1.66	3.02	
	**Total**	5	8	35	23	15	
**Spermatozoa concentration (millions/mL)**	**Mean change**	-35.89	-20.09		34	65.15	-15.02
	**SD change**	40.183	26.975		37.8	64.84	19.029
	**Total**	5	8		23	15	50
**Total sperm count (millions)**	**Mean change**	-107.15	-53.05	-105.7	137	169.85	
	**SD change**	116.136	74.908	111.547	167.6	160.65	
	**Total**	5	8	35	23	15	
**Initial forward motility (% of motile)**	**Mean change**	1.34	0.8		31.5	34.75	-14.9
	**SD change**	14.1921	12.29		15.72	21.71	16.108
	**Total**	5	8		23	15	50
**Spermatozoa morphology (% of normal)**	**Mean change**	12.58	-2.76		14.25	9.25	-10
	**SD change**	13.469	9.908		11.31	7.46	23.563
	**Total**	5	8		23	15	50
**Vitality (% of living)**	**Mean change**	-7.75	-15.35		50.75	48.5	
	**SD change**	13.3217	13.271		25.46	18.91	
	**Total**	5	8		23	15	
**Serum Testosterone (ng/dL)**	**Mean change**						
	**SD change**						
	**Total**						
**Patients returning to normal after 3 months of stoppage of HU**	**Event**						11
	**Total**						15

HU: hydroxyurea; SD: standard deviation; y: Years. Cells shaded in gray indicate that data is not available.

**Table 2:** Summary of studies in which female sickle cell anaemia patients were treated with hydroxyurea.

Study ID			Elchuri 2015			George 2022	Pecker 2020	Joseph 2023
Intervention			Supportive care	HU	BMT	HU	HU	HU
**Mean Age**	**(y)**		9.7			17	25.5	24.5
**Anti-Müllerian hormone**	**(ng/mL)**	**Mean**	2.394	1.876	0	2.07	2.09	1.31
		**SD**	1.498	2.156	0	1.4	1.5154	2.72
		**Total**	14	33	9	14	15	33
**Normal ovarian reserve**	**Follicles per-ovary**	**Event**	14	25	0		10	12
		**Total**	14	33	9		15	36
**Diminished ovarian reserve**	**Non-menopausal FSH**	**Event**	0	8	1	2	5	
		**Total**	14	33	9	14	15	
**Premature ovarian insufficiency**	**Menopausal FSH**	**Event**	0	0	8	5		
		**Total**	14	33	9	14		
**Spontaneous menarche**		**Event**	8	22	0			
		**Total**	14	33	9			
**Onset of menarche < 15 years of age**		**Event**	2	14				
		**Total**	14	33				
**Onset of menarche ≥ 15 years of age**		**Event**	4	6				
		**Total**	14	33				
**No menarche (Current age < 15 years of age)**		**Event**	3	7	6			
		**Total**	14	33	9			
**No menarche (Current age ≥ 15 years of age)**		**Event**	0	0	3			
		**Total**	14	33	9			

Bone marrow transplant; HU: Hydroxyurea; FSH: Follicle stimulating hormone; y: years. Cells shaded in gray indicate that data is not available.

### Quality assessment of studies

A meta-analysis was performed to report the outcomes of HU therapy on fertility parameters in SCD patients. This included information from four cohort studies for males encompassing 205 patients [25, 35, 39, 40] (Table 1) and four cohort studies on females assessing 149 individuals [41–43] (Table 2). Three of the male studies were deemed to be of high quality, with scores ≥ 7 on the Newcastle-Ottawa Scale [25, 39, 40] (NOS; Table 3). One study was deemed moderate quality due to the lack of follow-up [35]. Of the female studies, three were deemed high quality [41, 43]. The study by George and colleagues (2022) was deemed low quality due to limited external controls and comparison of cohorts [42]. Collectively, the available pooled data regarding female fertility outcomes were limited, but sufficient to perform a meta-analysis of AMH levels and the percentage of patients who had diminished ovarian reserve as a measure of the effects of HU on fertility. All four female studies used serum AMH as a measurement for ovarian reserve [41–43]. Additionally, two of the studies also tested for serum levels of Follicle-stimulating hormone (FSH) with one using serum FSH for the purpose of classifying women with premature ovarian failure once serum FSH levels are >40 IU/L [42, 43]. Although these studies did not report the phase of menstrual cycle at which the tests were performed, serum AMH levels do not differ across the menstrual cycle.

**Table 3:** Newcastle-Ottawa Scale (NOS) assessment of the quality of included studies.

Study ID		*Berthaut 2008	*Berthaut 2017	*Joseph 2021	*Sahoo 2017	^#^Elchuri 2015	^#^George 2022	^#^Pecker 2020	#Joseph 2023
Study Participants		35	44	15	100	56	14	46	33
**Selection**	**Representative of the exposed cohort**	*	*	*	*	*	*	*	*
	**Selection of external control**	0	0	*	0	*	0	*	*
	**Ascertainment of exposure**	*	*	*	*	*	*	*	*
	**Outcome of interest not present at the start of the study**	*	*	*	*	*	*	*	*
**Comparability of cohorts**	**Main Factor**	*	*	*	*	*	0	*	*
	**Additional factor**	*		*	*	*	0	*	*
	**Assessment of outcomes**	*	*	*	*	*	0	*	*
**Outcome**	**Sufficient follow up time**	*	*	*	0	*	*	*	*
	**Adequacy of follow up**	0	*	*	0	*	*	*	*
	**Total Score (/9)**	7	7	9	6	9	5	9	9
	**Average by Gender**	Female: 7.25				Male: 8.00			

Total scores ≥ 7 are deemed high quality with a low risk of bias. Total scores <5 were categorised as low quality with a high risk of inherent bias. Scores between these values were rated as moderate quality

*Male studies; ^#^Female studies.

### Volume of ejaculate (mL) in males studies

**Fig 2:**
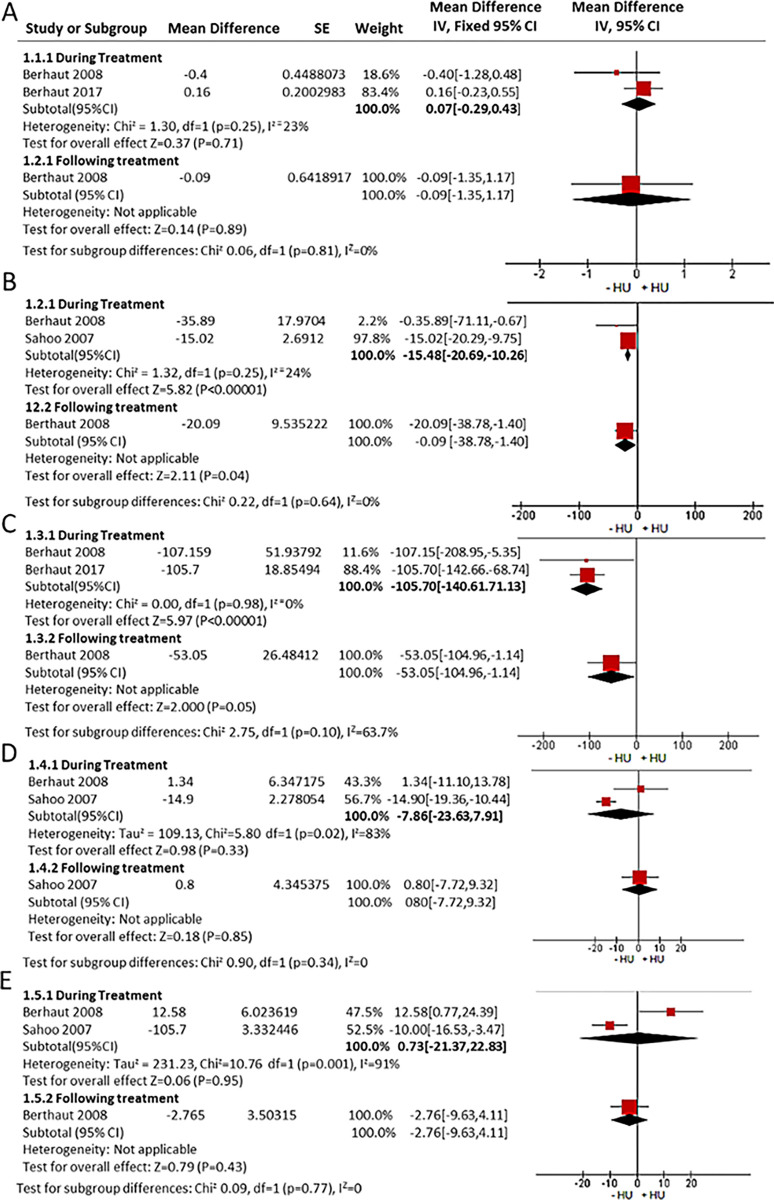
Forest plots showing the mean difference from baseline for the indicated male outcomes following HU treatment. **(A)** Volume of ejaculate (mL); **(B)** Spermatozoa concentration (millions/mL); **(C)** Total sperm count (millions), **(D)** Initial forward motility (% of motile sperm); **(E)** Spermatozoa morphology (% of normal).

Pooled data from male studies showed that the overall mean difference in the volume of ejaculate (mL) was not significantly impacted by HU treatment (MD = 0.07 mL; 95% CI: [-0.29, 0.43]; p = 0.71) and (MD = -0.09 mL; 95% CI: [-1.35, 1.17]; P = 0.89) respectively (Fig 2A). Table 3 shows the quality assessment of the included studies. The fixed effect model was used for these analyses.

### Spermatozoa concentration (millions/mL) in male studies

Pooled data from the mean difference from baseline showed that HU significantly reduced the concentration of spermatozoa during treatment (MD = -15.48 million/mL; 95% CI: [-20.69, -10.26]; p< 0.001). This did not recover following the cessation of HU treatment (MD = -20.09 million/mL; 95% CI: [-38.78, -1.40]; P = 0.04; Fig 2B). No overt indication of publication bias was observed with an average NOS score of 7.67 (Table 3).

### Total sperm count (millions) in male studies

The overall mean difference from baseline showed that HU significantly reduced the total sperm count during treatment, which did not recover following HU cessation (MD = -105.87 million; 95% CI: [-140.61, -71.13]; P< 0.001), and (MD = -53.05 million; 95% CI: [-104.96, -1.14]; P = 0.05), respectively (Fig 2C).

### Initial forward motility (% of motile) in male studies

The overall mean difference from baseline showed that HU did not significantly impact the forward motility of sperm during treatment (MD = -7.86% of motile; 95% CI: [-23.63, 7.91]; P = 0.33), or following cessation (MD = 0.80% of motile; 95% CI: [-38.78, -1.40]; P = 0.04), respectively. pooled studies were not homogeneous for those currently receiving HU therapy (P = 0.002; I^2^ = 83%, Fig 2D).

### Spermatozoa morphology (% of normal) in male studies

The overall mean difference from baseline showed that HU treatment did not significantly impact initial forward motility during- (MD = 0.73% of normal; 95% CI: [-21.37, 22.83]; P = 0.95) or after treatment (MD = -2.76% of normal; 95% CI: [-9.63, 4.11]; P = 0.43). Pooled studies were not homogeneous for the HU treatment subgroup (P = 0.001; I^2^ = 91%), Fig 2E).

### Anti-Müllerian hormone in female studies

**Fig 3:**
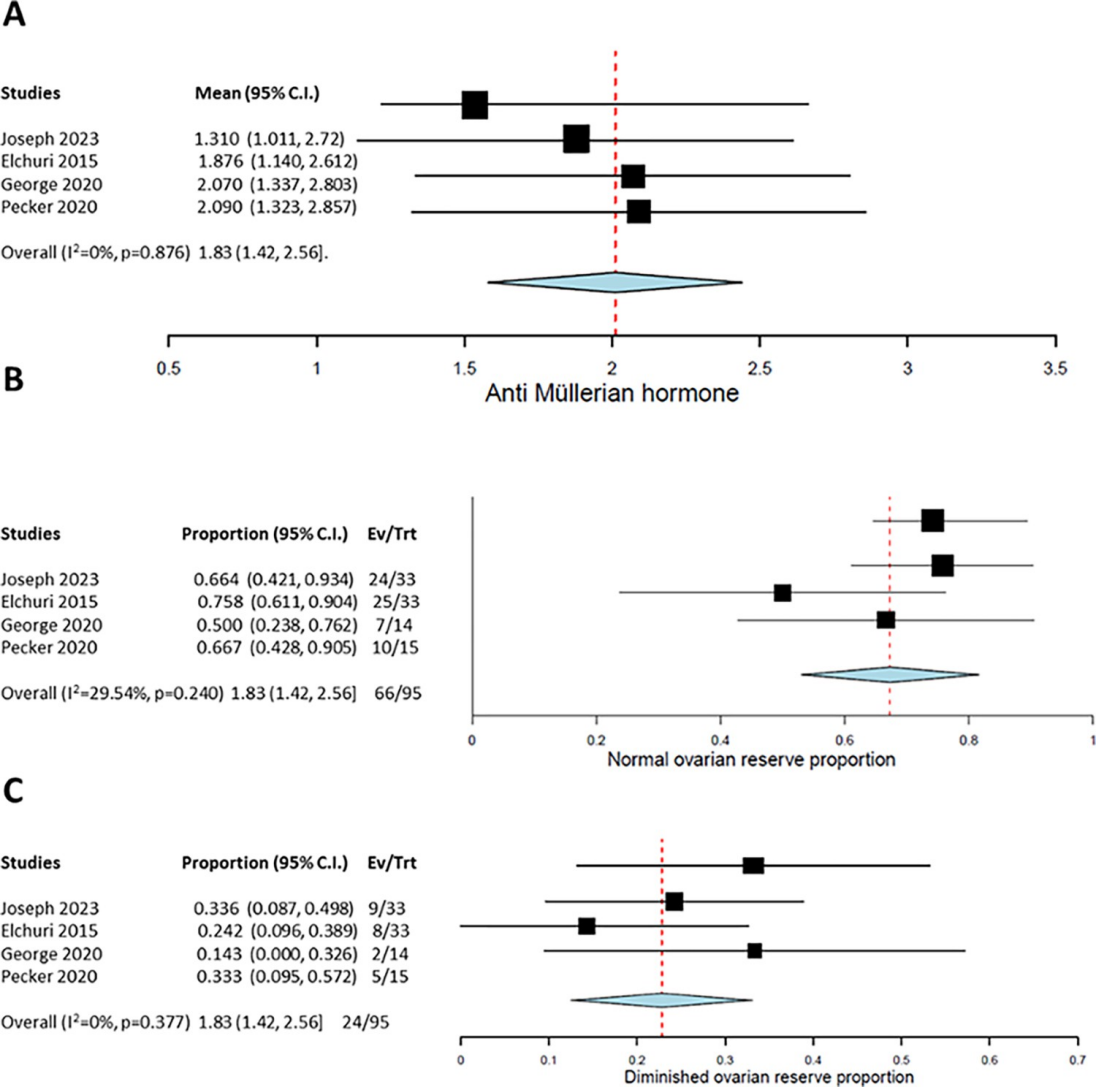
Forest plots of the major female outcomes following HU therapy. **(A)** Mean difference in anti-Müllerian hormone levels; **(B)** Proportion of patients with normal ovarian reserves; **(C)** Proportion of patients with diminished ovarian reserves.

The mean AMH in SCD patients at baseline is ~7.6 pmol/l compared with 13.4 pmol/l in healthy patients aged 35–36 years (p<0.001) [44]. HU treatment is also independently associated with low AMH values (beta = 0.001, 95% CI -0.002 to 0.000; P = 0.006) [41]. Our analyses supported these data, as SCD patients had a significantly higher chance of low AMH values compared to age-matched control groups (OR 2.6 (CI 1.1–6.5, P = 0.02). Three of the four studies were of high quality (Table 3). Using these data, a meta-analysis of proportions was performed using a DerSimonian-Liard meta-analysis model. Following HU treatment, pooled mean values of AMH were 1.83 (95% CI [1.42, 2.56]; Fig 3A) which were below the normal range (0.256–6.345) for the mean age of the cohort (mean age = 19.17 years; normal AMH values for 19–29 years (13.1–53.8). This suggested that HU is associated with reduced AMH levels in female SCD patients.

### Ovarian function

During and after HU treatment, a total of 72.2% of patients showed normal ovarian reserves (95% CI [42%, 89%]). The remainder (18.8%) showed diminished reserves (95% CI [11%, 43%]) Fig 3B, highlighting a significant negative impact on fertility in females.

## Discussion

Clinical records of patients with SCD (haemoglobin SS genotype) have identified gender-related differences in disease progression, independent of treatment modalities [45]. These include the frequency of pain crises, infections and cardiovascular issues. These are attributed to hormonal variations between males and females after puberty [46–49]. Gender is therefore a valuable factor in the risk stratification of SCD at diagnosis, with the possibility to guide therapeutic decisions [45].

HU has many characteristics of an ideal drug for SCD [10, 50–60], but concerns regarding adverse effects on fertility have persisted [25, 31, 35, 39–44, 61–64]. In this systematic review and meta-analysis, the impact of HU on fertility was comparable in male and female SCD patients. This was important to understand as differences in treatment responses and adverse events of drugs between genders are not uncommon and have been documented for chemotherapeutics, anti-depressants and irritable bowel syndrome medications [65].

Infertility is defined in as a failure to conceive after 12 months of unprotected heterosexual intercourse. Semen and ovarian reserve are used to assess fertility and to predict the success of fertility preserving interventions. Based on our findings and as a precaution, such interventions may be required for HU-treated SCD patients, inclusive of sperm and ovarian fertility preservation options (in patients of an applicable age). The pooled effects suggested that in males, HU negatively influenced the concentration of spermatozoa during treatment and after cessation. This led to diminished total sperm counts. In females, the pooled effects suggested that HU treatment impacted the mean AMH levels and normal ovarian reserves in some patients. This suggests that HU impacts seminal fluid parameters in males and reduces ovarian reserves to a significant level in SCD patients.

Fertility counselling for SCD patients involves complex decision-making and the discussion of side-effects. Key factors that should be addressed include methods for fertility preservation, pregnancy possibilities/outcomes and infertility treatment [66]. Despite the known risks of HU for fertility, assisted reproductive technologies remain largely unavailable to SCD patients [67]. Based on our pooled analyses, counsellor’s should work with both genders exposed to HU and fully inform them regarding its long-term impact. The importance of this is highlighted by the limited recovery observed upon the cessation of HU in the limited number of studies assessed.

To-date it has been unclear whether male and female patients have different risk factors that impact fertility following HU therapy. Our analyses most importantly highlights how education regarding common infertility risks factors must be addressed alongside disease- and treatment- associated fertility risks, and is of equal importance in both genders. Full education can avoid the possibility that adults with SCD refuse treatment due to infertility concerns [67–69].

### Study limitations

In males, prior to the introduction of HU treatment, patients had abnormal sperm values related to SCD itself. Similarly, female patients with SCD had lower levels of AMH in comparison to controls. This may have influenced the outcomes to HU therapy. In some studies, the possibility of determining whether HU was used at the time of AMH measurement were unclear. Subjects were therefore classed as receiving HU if they were “taking” or “ever took” the therapy. The number of relevant studies was also small (n = 4 males and n = 4 females), limiting heterogeneity. The actual probability of full recovery after HU therapy was not possible to document and requires more detailed investigation. Collectively, this highlights the need for long-term multi-centric studies using consistent HU doses and outcome assessments to fully understand the impact of HU in SCD patients.

### Conclusions

Fertility preservation remains an important consideration for individuals with SCD receiving HU, who may face reproductive challenges. Our findings suggest that the use of HU for SCD impacts seminal fluid parameters in males and diminishes AMH and ovarian reserves in females. Based on this finding, it is the opinion of the authors that fertility preservation counselling should be considered in patients with SCD in both genders of reproductive age prior to HU therapy. Increased knowledge of fertility in both males and females following the cessation of HU therapy is now urgently required given the limited recovery observed and lack of available data to fully document recovery. Longitudinal post-fertility care based on these findings should now be recommended to all HU treated SCD patients.

## Supporting information

S1 Checklist PRISMA 2020 checklist. (DOCX)
